# Concise Synthesis of Both Enantiomers of Pilocarpine

**DOI:** 10.3390/molecules26123676

**Published:** 2021-06-16

**Authors:** Theresa Schmidt, Niels Heise, Kurt Merzweiler, Hans-Peter Deigner, Ahmed Al-Harrasi, René Csuk

**Affiliations:** 1 Department of Organic Chemistry, Martin-Luther University Halle-Wittenberg, Kurt-Mothes Str. 2, D-06120 Halle (Saale), Germany; theresa.schmidt@chemie.uni-halle.de (T.S.); niels.heise@student.uni-halle.de (N.H.); 2 Department of Inorganic Chemistry, Martin-Luther University Halle-Wittenberg, Kurt-Mothes Str. 2, D-06120 Halle (Saale), Germany; kurt.merzweiler@chemie.uni-halle.de; 3 Medical and Life Science Faculty, Institute of Precision Medicine, Furtwangen University, Jakob-Kienzle, Str. 17, D-78054 Villingen–Schwenningen, Germany; dei@hs-furtwangen.de; 4 Chair of Oman’s Medicinal Plants and Marine Natural Products, University of Nizwa, P.O. Box 33, PC 616 Nizwa, Oman; aharrasi@unizwa.edu.com

**Keywords:** pilocarpine, enzymatic resolution, alkaloids

## Abstract

Furan-2-carboxylic acid was used as a starting material for the synthesis of dehydro-homopilopic acid. Esterification, hydrogenation and enzymatic hydrolysis followed by the reduction of Weinreb amides and a single-step attachment of a 1-methyl-imidazole residue allowed for the concise synthesis of both enantiomers of pilocarpine.

## 1. Introduction

**Figure 1:**
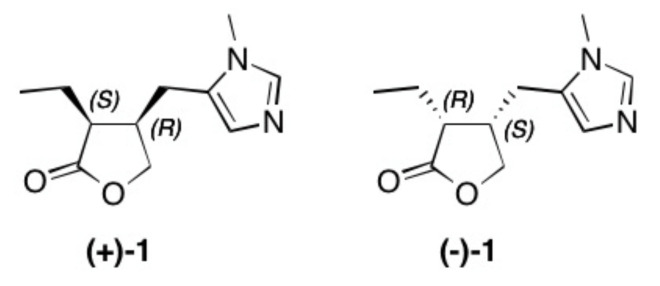
Structure of natural occurring pilocarpine **(+)-1** and its enantiomer **(–)-1**.

Pilocarpine **(+)-1** (Figure 1) is the main alkaloid of the South American tree *Pilocarpus jaborandi*, and it is usually isolated from its leaves. Pilocarpine is used as a cholinergic [1,2,3] parasympathomimetic [4,5,6,7,8], hidrotic and miotic agent [9,10]; in ophthalmology, it is used against glaucoma [11,12,13] and also acts as an antagonist [14,15,16] to atropine and as an inhibitor of the enzyme carbonic anhydrase II [17,18,19]. Moreover, a deficiency of carbonic anhydrase in humans is linked to osteopetrosis; thereby, osteoclasts are unable to perform bone resorption in a normal way [20]. Recently, some imidazolic alkaloids have been identified as promising targets as possible inhibitors of the main protease of SARS-CoV-2 [21]. The compound was first isolated by Petit and Polonovski [22,23] as well as by Gerrard [24] and Hardy [25], its structure was determined based on the studies of Jowett [26] and Zav’yalov [27], and the absolute configuration of this alkaloid was determined by Hill and Barcza [28]. A biosynthetic pathway has been suggested in 2015 [29].

However, the content of **(+)-1** is very low in the leaves of different species of trees of the genus *Pilocarpus*; it ranges from 0.12–0.6% in *P. racemosous* [30,31,32] and from 0.45% in *P. microphyllus* [33,34,35] and *P. pennatifolius* [36,37] to 0.8% in *P. jaborandi* [38,39]. Due to the low content of the alkaloid in the plant material as well as a laborious extraction procedure [40], a number of syntheses have been described for this interesting molecule [41,42,43,44,45,46,47,48,49,50,51,52,53,54,55,56,57]. Many of them, however, are multi-step and laborious, often of low yield, or they yield a racemic product. In the context of our own studies on inhibitors of carbonic anhydrases, we were interested in an efficient access to **(+)-1** but also its enantiomer **(–)-1**. This approach uses, in part, a chemoenzymatic strategy that we have previously [42] used to synthesize natural occurring **(+)-1**.

## 2. Results

In our own synthetic strategy, the introduction of the 1-methylimidazole residue should be the last step of the synthesis, since this residue—as previously shown [42] —can be introduced in a single step and under very mild conditions using a Leusen imidazole synthesis [58,59,60,61]. However, a prerequisite for carrying out this planned synthetic sequence is that (homo)-pilopaldehyde or (homo)-pilopic acid must be present in pure enantiomeric and diastereomeric forms. Furthermore, these intermediates should be accessible in high yields. Since we were interested in the synthesis of both pilocarpine enantiomers, a synthesis to racemic (homo)-pilopic acid with a subsequent separation of the enantiomers seemed to be a reasonable strategy. A good starting material should be readily accessible furan-2-carboxylic acid (**2**).

**Scheme 1:**
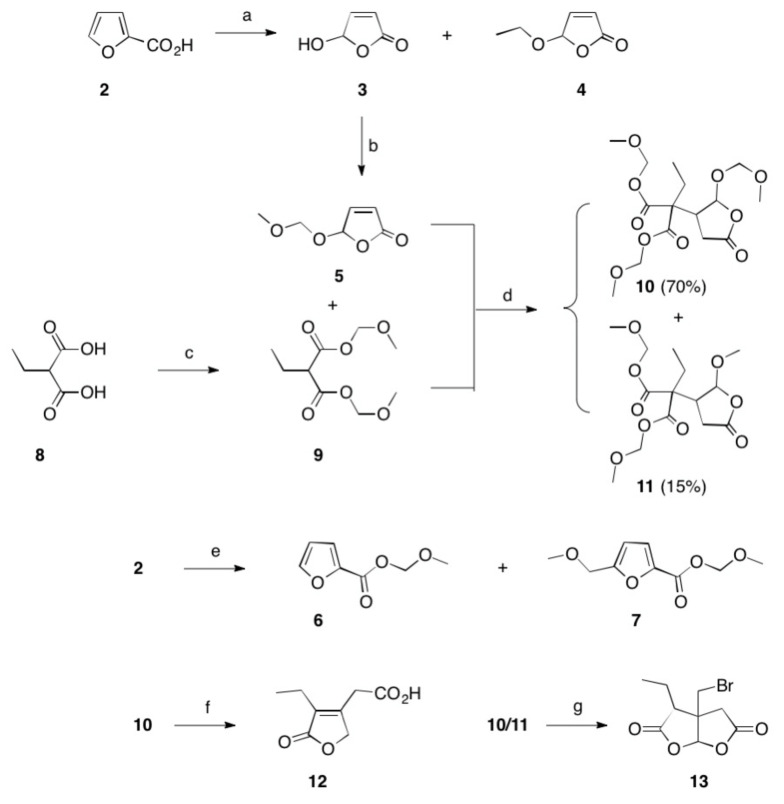
Reactions and conditions: (**a**) hν, Bengal rosa, 8 h, 20 °C, 76% (of **3**) and 5% (of **4**); (**b**) CH_2_(OCH_3_)_2_, P_4_O_10_, DCM, 20 °C, 5 h, 98%; (**c**) CH_2_(OCH_3_)_2_, P_4_O_10_, DCM, 20 °C, 5 h, 99%; (**d**) THF, Na, 25 °C, 15 h, 72%; (**e**) CH_2_(OCH_3_)_2_, P_4_O_10_, DCM, 20 °C, 5 h, 77% (of **6**) and 19% (of **7**); (**f**) HBr, reflux, 2 d, 83%; (**g**) HBr, reflux, 4 d, 4%.

Thus, **2** (Scheme 1) was photo-oxidized [62] in the presence of oxygen and the sensitizer bengal rosa via an intermediately formed hydroperoxide to yield **3**. Thereby, **4** was formed as the by-product (by acetalisation). Since acetals are easy to introduce on the one hand, but also easy to cleave off on the other, they should be our preferred protecting groups in the next steps. Hereby, the methoxymethylation of the free hydroxyl groups using formaldehyde dimethyl acetal seems particularly suitable, since these reactions are known to proceed mostly with very high yields. In previous experiments, furfural [63,64,65] had been oxidized as an alternative to **2**, but polymerization reactions were observed to a high extent during its photo-oxidation.

**Figure 2:**
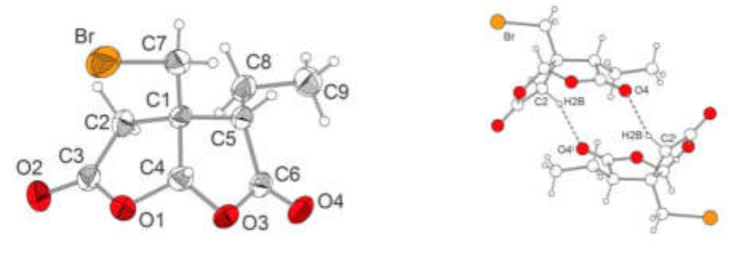
Molecular structure of compound **13** in the crystal. Thermal ellipsoids with a 50% probability. H atoms with arbitrary radius. C–H^…^O hydrogen bonds between two symmetrically related enantiomeric molecules. Selected bond lengths (pm) and angles (°): C(1)–C(2) 154.1(4), C(1)–C(4) 154.6(4), C(1)–C(5) 154.6(4), C(1)–C(7) 152.1(4), C(2)–C(3) 149.6(4), C(3)–O(1) 135.9(4), C(3)–O(2) 119.3(4), C(4)–O(1) 141.7(3), C(4)–O(3) 141.7(4), C(5)–C(6) 151.5(4), C(5)–C(8) 153.0(4), C(6)–O(3) 136.4(4), C(6)–O(4) 119.4(4), C(7)–Br 195.4(3), C(8)–C(9) 151.9(4), C(2)–C(1)–C(4) 102.1(2), C(3)–C(2)–C(1) 105.7(3), O(1)–C(3)–C(2) 110.5(2), C(3)–O(1)–C(4) 111.3(2), O(1)–C(4)–C(1) 108.6(2), C(5)–C(1)–C(4) 103.0(2), C(6)–C(5)–C(1) 103.9(2), O(3)–C(6)–C(5) 110.3(2), C(6)–O(3)–C(4) 111.8(2), O(3)–C(4)–C(1) 107.6(2), C(1)–C(7)–Br 112.4(2). Hydrogen bond C(2)–H(2B)–O4^i^: C(2)^…^O(4)^i^ 316.7, C(2)–H(2B)–O(4)^i^ 125.98; symmetry operator i: –x, -y+1, -z+1.

The reaction of **3** with formaldehyde dimethyl acetal in the presence of solid P_4_O_10_ [66] produced an almost quantitative yield of methoxymethylated **5**. Interestingly enough, applying the same conditions from **2**, ester **6** was formed together with **7** as a minor byproduct. These reaction conditions can therefore be used not only to very efficiently introduce a protecting group on a hydroxyl group but also to protect the carboxylic acids. 2-Ethylmalonic acid (**8**) was also neatly transformed into its bis(methoxymethylester) **9**. The Michael addition of the latter with **5** produced **10** in a 70% isolated yield and 15% of **11** as a by-product; the latter product was formed by transacetalisation. A Stobbe condensation [47] of **10** for 2 days produced dehydrohomopilopic acid (**12**) together with traces of a side product **13**. To elucidate the formation and structure of **13**, a mixture of **10** and **11** was heated under reflux with aqueous HBr (48%) for 4 days, and **13** was obtained as colorless crystals. The results from the mass spectrometry showed **13** to hold a bromine substituent, and from the interpretation of ^1^H and the ^13^C-NMR spectra, **13** was assigned the structure of a 3a-(bromomethyl)-3-ethyl-dihydro-[2,3-b]furan-(3H, 4H)-2,5-dione. For verification of this structure, suitable crystals were grown that were subjected to a single crystal X-ray analysis, whose results are depicted in Table 1 and Figure 2. The formation of **13** remains unclear but should start by a cleavage of the ester moieties of **11** by HBr followed by a decarboxylation of the substituted malonic acid and intramolecular lactonization.

Details of the data collection and refinement of the crystal structure of compound **13** are collected in Table 1. Compound **13** (Figure 2) crystallizes in the monoclinic space group P2_1_/n with four formula units per unit cell. Due to its crystallographic symmetry, the crystal structure contains both enantiomers of compound **13** as racemate. Moreover, the enantiomeric pairs are linked by weak C–H^…^O hydrogen bonds (Figure 2, right). Compound **13** exhibits C–C, C–O and C–Br bond lengths that are in the expected range. Both the lactone rings C1–C2–C3–O1–C4 and C1–C5–C6–O3–C4 exhibit envelope conformation with the central C1 atom at the flap position. However, C1 differs only 8.2 and 11.1 pm, respectively, from the corresponding mean planes. The interplanar angle between both lactone rings is 64.4°.

**Scheme 2:**
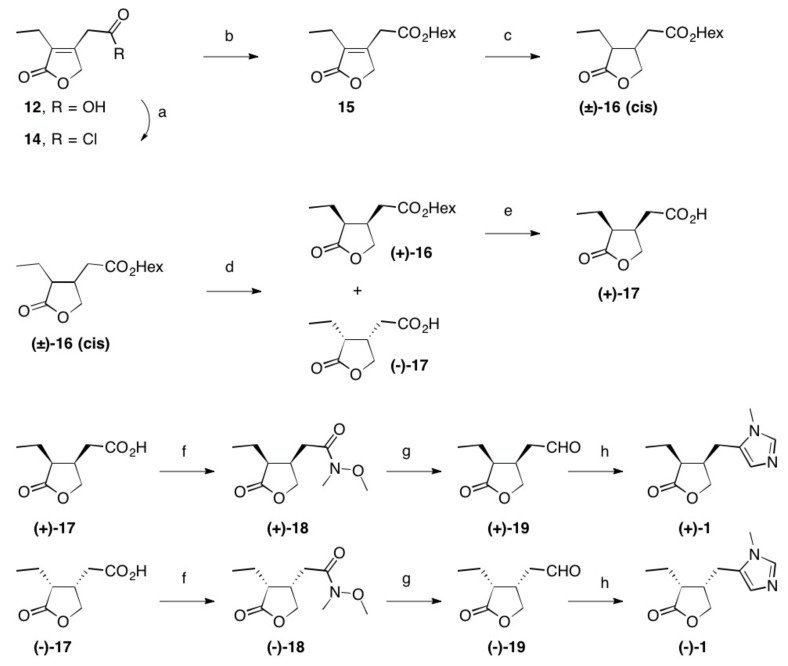
Reactions and conditions: (**a**) SOCl_2_, reflux, 3 h, quant.; (**b**) Hex-OH, reflux, 16 h, 98%; (**c**) Rh/Al_2_O_3_, H_2_ (1 at), THF, 5 d, quant.; (**d**) Lipase PS, pH = 7.0, 2 d, 22 °C, 48% (of **(±)-16**) and 42% (of **(–)-17**); (**e**) PLE, pH = 7.0, 22 °C, 2 d, 96%; (**f**) *N*-methylmorpholine, ^i^Bu-chloroformate, *N*,*O*-dimethylhydroxylamine hydrochloride, 23 °C, 1 d, 84% (of **(+)-18**) and 85% of **(–)-18**); (**g**) LiAlH_4_, Et_2_O, 23 °C, 30 min, 95% (of **(+)-19**) and 95% of **(–)-19**; (**h**) CH_3_NH_2_, TosMic, DCM, benzene, NEt_3_, 7 d, 23 °C, 59% (of **(+1)-1** and 60% of **(–)-1**; Hex stands for *n*-hexyl.

It seems convenient to perform a resolution of the racemate at this stage. An enantioselective hydrolysis of an ester with a suitable enzyme appeared to be particularly attractive. Thus, **12** was activated using thionyl chloride (Scheme 2) to afford in situ the acid chloride **14**, the reaction of which with *n*-hexanol produced ester **15.**

The hydrogenation of **15** using Rh/Al_2_O_3_ for 5 days at atmospheric pressure produced cis-configurated **(±)-16**; its cis-configuration was confirmed by NMR. This ester was reacted under pH-stat conditions at pH=7 with various hydrolytic enzymes (acylase from *Aspergillus* sp., chymotrypsin A4, Fungamyl 800L^®^, Lipase from *Candida rugosa*, Lipase from *Candida lipolytica*, Lipase from *Mucor miehei, Penicillium roqueforti, Rhizopus arrhizus* and from *Rh. delemar*, lipase type II from porcine pancreas, lipase AY (Amano), lipase F AP15 (Amano), lipase M10 (Amano), Lipase OF (Amano), lipase P (Amano), lipase PS (Amano), lipolase 100L^®^, lipozyme^®^, novozym 450^®^, pancreatin, pronase, proteases XXIII, XIV, XXI, pig liver esterase (PLE), subitilisin, Thermo Cat E 003 to 015^®^). Thereby, hydrolysis by lipase PS of Amano proved suitable, and **(+)-16** and **(–)-17** were each obtained in excellent yields. Their enantiomeric purity was determined to be > 99% by HPLC using chiral phases (Chiralcel OC and Chiralpak AS).

While hydrolysis of **(+)-16** using aq. NaOH resulted in partial isomerization, enzymatic hydrolysis using pig liver esterase (PLE) at pH = 7 readily afforded **(+)-17** with an enantiomeric excess of ee > 99.

Thus, both enantiomers of homopilopic acid **17** are readily accessible in pure enantiomeric form. For the synthesis of **(–)-1** and **(+)-1**, **17** was reacted with *N*-methylmorpholine, isobutyl chloroformate and *N*,*O*-dimethylhydroxylamine hydrochloride [67,68,69,70,71,72] to obtain the Weinreb acetamides **(–)-18** and **(–)-18** in 85% and 84% yields, respectively. Their reduction with LiAlH_4_ produced aldehydes [44,56] **(–)-19** and **(+)-19**, which, after reaction with methylamine/*p*-tosylmethylisocyanide (TosMic) [58,59] in the presence of triethylamine, produced **(–)-1** and **(+)-1**. Their enantiomeric purity was determined via HPLC using a chiral phase (Chiralcel OC) with >99% in each case.

## 3. Conclusions

Furan-2-carboxylic acid served as a valuable starting material for the straightforward synthesis of dehydro-homopilopic acid. Esterification, hydrogenation and enzymatic hydrolysis followed by the reduction of Weinreb amides and the single-step attachment of a 1-methyl-imidazole residue allowed for the convenient synthesis of both enantiomers of pilocarpine in good overall yields.

## 4. Experimental

NMR spectra were recorded using the Varian spectrometers (Darmstadt, Germany) DD2 and VNMRS (400 and 500 MHz, respectively). MS spectra were taken on a Advion expression^L^ CMS mass spectrometer (Ithaca, NY, USA); positive ion polarity mode, solvent: methanol, solvent flow: 0.2 mL/min, spray voltage: 5.17 kV, source voltage: 77 V, APCI corona discharge: 4.2 μA, capillary temperature: 250 °C, capillary voltage: 180 V, sheath gas: N_2_). Thin-layer chromatography was performed on pre-coated silica gel plates supplied by Macherey-Nagel (Düren, Germany). IR spectra were recorded on a Spectrum 1000 FT-IR-spectrometer from Perkin Elmer (Rodgau, Germany). The UV/Vis-spectra were recorded on a Lambda 14 spectrometer from Perkin Elmer (Rodgau, Germany); optical rotations were measured at 20 °C using a JASCO-P2000 instrument (JASCO Germany GmbH, Pfungstadt, Germany) The melting points were determined using the Leica hot stage microscope Galen III (Leica Biosystems, Nussloch, Germany) and are uncorrected. The solvents were dried according to usual procedures. Microanalyses were performed with an Elementar Vario EL (CHNS) instrument (Elementar Analysensysteme GmbH, Elementar-Straße 1, D-63505, Langenselbold, Germany). The crystal structure of compound **13** was solved by direct methods (SHELXS) and refined with the SHELXL (2008) program [73]. OLEX2 (2021) was used as graphical user interface [74]. The hydrogen atoms were positioned geometrically using a riding model. The crystal structure drawings were generated with DIAMOND (4.4.0) [75]. CCDC 2086056 contains the supplementary crystallographic data for this paper. These data can be obtained free of charge from The Cambridge Crystallographic Data Centre via www.ccdc.cam.ac.uk/structures.

### 4.1. 5-Hydroxyfuran-2(5H)-one (3) and 5-ethoxyfuran-2(5H)-one (4)

Oxygen was bubbled through a solution of **2** (10.0 g, 89.2 mmol) and Bengal rosa (350 mg) in ethanol (600 mL); the reaction mixture was irradiated at 20 °C (Heraeus middle pressure Hg lamp; 450 Watt) for 8 h. The solution was filtered through a pad of activated charcoal, and the solvents were removed under diminished pressure to yield a crude product that was subjected to chromatography (SiO_2_, *n*-hexane/ethyl acetate 5:1 → 3:1→ 2:1 → 1:1) to yield **3** (6.8 g, 76%) and **4** (517 mg, 5%); the unreacted starting material **2** (1.32 g, 13%) was recovered.

Data for **3**: colorless crystals, m.p. 51–53 °C (lit.: [62] 51–54 °C); R_f_ = 0.17 (*n*-hexane/ethyl acetate, 1:1); IR (KBr): ν = 3314brm, 3113m, 2924w, 1794m, 1760s, 1738s, 1733s, 1611w, 1446w, 1329m, 1282m, 1183m, 1131s, 1084m, 996s, 917m, 898m, 828s, 822s, 819m, 791m, 703m, 686m, 668m, 656m, 648m, 639m, 634m and 619m cm^−1^; ^1^H-NMR (400 MHz, CDCl_3_): δ = 7.34–7.24 (m, 1H, 4-H), 6.27–6.13 (m, 2H, 3-H, 5-H), 5.58–5.22 (br s, 1H, OH) ppm; ^13^C-NMR (100 MHz, CDCl_3_): δ = 171.7 (C-2), 152.3 (C-4), 124.5 (C-3) and 98.9 (C-5) ppm; MS (ESI, MeOH): *m/z* = 101.0 (60%, [M+H]^+^); analysis calcd. for C_4_H_4_O_3_ (100.07): C 48.01, H 4.03; found: C 47.87, H 4.19.

Data for **4**: off-white oil; b.p. 84–89 °C/8 mm (lit.: [47] 85–89 °C/mm); R_f_ = 0.61 (*n*-hexane/ethyl acetate, 1:1); IR (film): ν = 3105w, 2981m, 2934m, 1793s, 1762s, 1614w, 1445w, 1376m, 1350m, 1137s, 1080m, 1040m, 1010s, 960m, 935m, 894m, 818m, 790m, 700m and 687m, cm^−1^; ^1^H-NMR (400 MHz, CDCl_3_): δ = 7.23 (dd, *J* = 5.7, 1.1 Hz, 1H, 4-H), 6.23 (dd, *J* = 5.7, 1.2 Hz, 1H, 3-H), 5.95 (dd, *J* = 1.2, 1.1 Hz, 1H, 5-H), 4.00–3.70 (m, 2H, 6-H) and 1.29 (t, *J* = 7.1 Hz, 3H, 7-H) ppm; ^13^C-NMR (100 MHz, CDCl_3_): δ = 170.6 (C-2), 150.5 (C-4), 125.0 (C-3), 103.3 (C-5), 66.2 (C-6) and 15.0 (C-7) ppm; MS (ESI, MeOH): *m/z* = 129.1 (25%, [M+H]^+^); analysis calcd. for C_6_H_8_O_3_ (128.13): C 56.24, H 6.29; found: C 56.01, H 6.44.

### 4.2. 5-(Methoxymethoxy)-furan-2(5H)-one (5)

P_4_O_10_ (10 × 2.0 g, in 30 min intervals) was added to a solution of **3** (4.0 g, 40 mmol) in formaldehyde dimethyl acetal (200 mL, 2.2 mol) and dry DCM (100 mL). The solvents were decanted, and the residue was extracted with diethyl ether (3 × 200 mL). The combined organic phases were washed with a saturated solution of NaHCO_3_, brine, and dried (MgSO_4_). The solvents were removed, and pure **5** (4.6 g, 98%) was obtained as a colorless oil pure enough for the next step; an analytical sample showed R_f_ = 0.28 (*n*-hexane/ethyl acetate, 3:1); IR (film): ν = 2960m, 2830w, 1794s, 1762s, 1654w, 1612w, 1560w, 1507w, 1370m, 1328m, 1220m, 1155s, 1125s, 1074s, 996s and 697m cm^−1^; ^1^H-NMR (400 MHz, CDCl_3_): δ = 7.28 (dd, *J* = 5.7, 1.1 Hz, 4-H), 6.24 (dd, *J* = 5.7, 1.2 Hz, 1H, 3-H), 6.15 (dd, *J* = 1.2, 1.1 Hz, 1H, 5-H), 4.99 (d, *J* = 6.6 Hz, 1H, 6-H_a_), 4.77 (d, *J* = 6.6 Hz, 1H, 6-H_b_) and 3.47 (s, 3H, 7-H) ppm; ^13^C-NMR (100 MHz, CDCl_3_): δ = 170.1 (C-2), 150.6 (C-4), 124.5 (C-3), 99.5 (C-5), 95.1 (C-6) and 56.4 (C-7) ppm; MS (ESI, MeOH): *m/z* = 145.2 (32%, [M+H]^+^); analysis calcd. for C_6_H_8_O_4_ (144.13): C 50.00, H 5.59; found: C 49.88, H 5.73.

### 4.3. Methoxymethyl Furan-2-carboxylate (6) and Methoxymethyl 5-(methoxymethyl)furan-2-carboxylate (7)

Following the procedure given for the synthesis of **5**, from **2** (3.0 g), formaldehyde dimethylacetal (100 mL, 1132 mmol) and P_4_O_10_, **6** (3.2 g, 77%) and **7** (1.0 g, 19%) were obtained each as a colorless oil.

Data for **6**: R_f_ = 0.79 (*n*-hexane/ethyl acetate, 1:1); IR (film): ν = 3140w, 2960m, 2835w, 1730s, 1642w, 1580m, 1570m, 21475s, 1410m, 1390m, 1298s, 1231m, 1215s, 1162s, 1119s, 1070s, 1015s, 932s, 905s, 885s and 762s cm^−1^; ^1^H-NMR (400 MHz, CDCl_3_): δ = 7.60 (dd, *J* = 1.7, 0.9 Hz, 1H, 5-H), 7.25 (dd, *J* = 3.5, 0.9 Hz, 1H, 3-H), 6.55 (dd, *J* = 3.5, 1.7 Hz, 1H, 4-H), 5.45 (s, 2H, 7-H) and 3.55 (s, 3H, 8-H) ppm; ^13^C-NMR (100 MHz, CDCl_3_): δ = 158.1 (C-5), 146.8 (C-5), 144.3 (C-2), 118.7 (C-3), 110.0 (C-4), 90.8 (C-7) and 57.8 (C-8) ppm; MS (ESI, MeOH): *m/z* = 157.2 (82%), [M+H]^+^); analysis calcd. for C_7_H_8_O_4_ (156.14): C 53.85, H 5.16; found: C 53.70, H 5.33.

Data for **7**; R_f_ = 0.65 (*n*-hexane/ethyl acetate, 1:1); IR (film): ν = 3127w, 2935m, 2829m, 1795w, 1728s, 1640w, 1595w, 1524m, 1468m, 1452m, 1405m, 1370m, 1300s, 1205s, 1165s, 1130s, 1081s, 1022m, 988m, 968m, 945s and 906s cm^−1^; ^1^H-NMR (400 MHz, CDCl_3_): δ = 7.21 (d, *J* = 3.4 Hz, 1H, 3-H), 6.46 (d, *J* = 3.4 Hz, 1H, 4-H), 5.45 (s, 2H, 9-H), 4.67 (s, 2H, 6-H), 3.53 (s, 3H, 7-H) and 3.41 (s, 3H, 10-H) ppm; ^13^C-NMR (100 MHz, CDCl_3_): δ = 157.8 (C-5), 156.5 (C-8), 143.8 (C-2), 119.3 (d, C-3), 110.6 (C-4), 90.6 (C-9), 66.4 (C-6), 58.8 (C-7) and 57.8 (C-10) ppm; MS (ESI, MeOH): *m/z* = 201.1 (64%, [M+H]^+^); analysis calcd. for C_9_H_12_O_5_ (200.19): C 54.00, H 6.04; found: C 53.89, H 6.21.

### 4.4. Bis(methoxymethyl) Ethylpropanedioate (9)

Following the procedure given for the synthesis of **5**, from ethylmalonic acid (**8**, 3.0 g, 22.7 mmol), formaldehyde dimethyl acetal (60 mL, 680 mmol) and P_4_O_10_, **9** (4.95 g, 99%) was obtained as a colorless oil (pure enough for the next step); an analytical sample showed R_f_ = 0.83 (*n*-hexane/ethyl acetate, 1:1); IR (film): ν = 3636w, 2970s, 2833m, 2087w, 1736s, 1630w, 1546w, 1463s, 1406m, 21462s, 1071s, 1036s and 958s cm^−1^; ^1^H-NMR (400 MHz, CDCl_3_): δ = 5.30 and 5.29 (each s, 4H, 6-H, 8-H), 3.48 (s, 6H, 7-H and 9-H), 3.37 (virt t, *J* = 7.5 Hz, 1H, 2-H), 2.00 (virt dq, *J* = 7.5, 7.5 Hz, 2H, 4-H) and 1.02 (t, *J* = 7.5, 3H, 5-H) ppm; ^13^C-NMR (100 MHz, CDCl_3_): δ = 168.6 (C-1 and C-3), 91.0 (C-6 and C-8), 57.7 (C-2), 22.1 (C-4), 14.0 (C-7) and 11.8 (C-5) ppm; MS (ESI, MeOH): *m/z* = 221.3 (17%, [M+H]^+^); analysis calcd for C_9_H_16_O_6_ (220.22): C 49.09, H 7.32; found: C 49.00, H 7.49.

### 4.5. Bis(methoxymethyl) Ethyl-[2-(methoxymethyl)-5-oxooxolan-3-yl] propanedioate (10) and Bis(methoxymethyl) ethyl-(2-methoxy-5-oxooxolan-3-yl) propanedioate (11)

Metallic sodium (2.0 g, 8.7 mmol, 31 mol%) was added to a solution of **5** (4.0 g, 27.8 mmol) and **9** (8.0 g, 36.2 mmol) in dry THF (200 mL) at -45 °C under argon, and the mixture was allowed to warm to -15 °C. Careful control of the temperature is mandatory, and the mixture was stirred between 5–15 °C until all of the sodium was dissolved. Stirring at 25 °C was continued for an additional 15 h. The usual aqueous work-up followed by chromatography (SiO_2_, *n*-hexane/ethyl acetate, 10:1 → 7:1 → 5:1) produced **10** (7.25 g, 72%) and **11** (1.4 g, 15%).

Data for **10**: colorless oil; R_f_ = 0.64 (*n*-hexane/ethyl acetate, 1:1); IR (film): ν = 3555w, 2966m, 2835w, 1790s, 1731s, 1468m, 1455m, 1405m, 1307m, 1213s, 1167s, 1080s, 1008s, 965s and 880s cm^−1^; ^1^H-NMR (400 MHz, CDCl_3_): δ = 5.77 (d, *J* = 1.6 Hz, 5-H), 5.39 (d, *J* = 5.9 Hz, 1H, 13-H_a_), 5.27 (d, *J* = 5.9 Hz, 1H, 15-H_a_), 5.21 (d, *J* = 5.9 Hz, 1H, 15-H_b_), 5.18 (d, *J* = 5.9 Hz, 13-H_b_), 4.92 (d, *J* = 6.7 Hz, 1H, 6-H_a_), 4.65 (d, *J* = 6.7 Hz, 1H, 6-H_b_), 3.48 (s, 6H, 14-H and 16-H), 3.45 (s, 3H, 7-H), 3.07 (ddd, *J* = 9.6, 3.0, 1.6 Hz, 1H, 4-H), 2.94 (dd, *J* = 18.3, 9.6 Hz, 3-H_a_), 2.62 (dd, *J* = 18.3, 3.0 Hz, 1H, 3-H_b_), 2.08 (q, *J* = 7.5 Hz, 1H, 11-H) and 0.96 (t, *J* = 7.5 Hz, 3H, 12-H) ppm; ^13^C-NMR (100 MHz, CDCl_3_): δ = 174.9 (C-2), 169.2 and 169.0 (C-7 and C-9), 106.0 (C-5), 91.9 (C-12 and C-14), 59.2 (C-8), 58.1 (C-13 and C-15), 56.9 (C-6), 43.9 (C-4), 30.4 (C-3), 26.4 (C-10), 14.0 (C-6) and 8.7 (C-11) ppm; MS (ESI, MeOH): *m/z* = 365.4 (49%, [M+H]^+^); analysis calcd. for C_15_H_24_O_10_ (364.35): C 49.45, H 6.64; found: 49.17, H 6.83.

Data for **11**: colorless oil; R_f_ = 0.65; (*n*-hexane/ethyl acetate, 1:1); IR (film): ν = 2948s, 2835m, 2088w, 1770s, 1740s, 1730s, 1640w, 1548w, 1465m, 1405m, 1455m, 1415m, 1390m, 1358m, 1310m, 1240s, 1210s, 1168s, 1115s, 1010s, 980s, 941s, 880s, 802m and 702m cm-1; ^1^H-NMR (400 MHz, CDCl_3_): δ = 5.40–5.25 (m, 4H, 12-H and 14-H), 5.31 (d, *J* = 4.7 Hz, 1H, 5-H), 3.50 (s, 3H, 6-H), 3.50 (s, 6H, 13-H, 15-H), 3.02 (ddd, *J* = 10.1, 4.7, 2.8 Hz, 1H, 4-H), 2.95 (dd, *J* = 17.3, 10.1 Hz, 1H, 3-Ha), 2.65 (dd, *J* = 17.3, 2.8 Hz, 1H, 3-Hb), 2.05 (q, *J* = 7.5 Hz, 2H, 10-H) and 0.95 (t, *J* = 7.5 Hz, 3H, 11-H) ppm; ^13^C-NMR (100 MHz, CDCl_3_): δ = 174.9 (C-2), 169.2 and 169.0 (C-7 and C-9), 106.0 (C-5), 91.9 (C-12 and C-14), 59.2 (C-8), 58.1 (C-13 and C-15), 56.9 (C-6), 44.0 (C-4), 30.4 (C-3), 26.4 (C-10) and 8.7 (C-11) ppm; MS (ESI, MeOH): *m/z* = 335.3 (48%, [M+H]^+^); analysis calcd. for C_14_H_22_O_9_ (334.32): C 50.30, H 6.63; found: 50.04, H 6.87.

### 4.6. (4-Ethyl-5-oxo-2,5-dihydrofuran-3-yl) Acetic Acid (12, Dehydro-homopilopic acid)

A solution of **10** (3.0 g, 8.23 mmol) was heated under reflux for 2 days in an aqueous solution of HBr (48%, 20 mL) followed by a continuous extraction with diethyl ether (3 × 50 mL, Kutscher–Steudel apparatus). The organic phase was dried (MgSO_4_), the solvent removed under diminished pressure, and the residue subjected to chromatography (SiO_2_, *n*-hexane/ethyl acetate, 2:1 → 1:1 → 0:1; then methanol/ethyl acetate 1:10 → 1:5) to yield **12** (1.16 g, 83%) as a colorless oil; b.p. 170 °C/3.10^−2^ mbar (lit.: [76] 172–173.5 °C/0.5 Torr); R_f_ = 0.42 (methanol/ethyl acetate, 1:1); IR (film): ν = 3507brw, 2976s, 2939s, 2880m, 1732s, 1673m, 1446m, 1380m, 1348m, 1200s, 1177s, 1110m, 1159m, 1035s, 950m and 776m cm^−1^; ^1^H-NMR (400 MHz, CDCl_3_): δ = 8.91 (br s, 1H, CO2H), 4.84 (d, *J* = 1.0 Hz, 2H, 5-H_a,b_), 3.54 (s, 2H, 8-H), 2.33 (q, *J* = 7.6 Hz, 6-H) and 1.11 (t, *J* = 7.6 Hz, 3H, 7-H) ppm; ^13^C-NMR (100 MHz, CDCl_3_): δ = 174.1 (s, C-9), 172.9 (C-2), 150.3 (C-4), 131.8 (C-3), 71.5 (C-5), 32.3 (C-8), 17.1 (C-6) and 12.5 (C-7) ppm; MS (ESI, MS): *m/z* = 171.3 (44%, [M+H]^+^); analysis calcd. for C_8_H_10_O_4_ (170.16): C 56.47, H 5.92; found: C 56.18, H 6.13.

### 4.7. 3a-(Bromomethyl)-3-Ethyl-dihydro-[2,3-b]furan-(3H, 4H)-2,5-dione (13)

A solution of **10** and **11** (ratio 1:5, 15.0 g) in aqu. HBr (48%, 200 mL) was heated under reflux for 4 days followed by an extraction for 1 day (3 × 150 mL, Kutscher–Steudel apparatus) and chromatography (SiO_2_, *n*-hexane/ethyl acetate, 1:1) to afford **13** (350 mg, 4%) as colorless crystals; m.p. 132–134 °C; R_f_ = 0.45 (*n*-hexane/ethyl acetate 1:1); IR (KBr): ν = 3560m, 2005s, 2975s, 2956m, 2940s, 2881m, 1784s, 1461s, 1442m, 1402s, 1370s, 1354s, 1330s, 1295s, 1274s, 1255m, 1229m, 1188s, 1153s, 1138s, 1095s, 1068s, 1042s, 1011s, 941s, 917s, 883s, 867s, 851s, 780m, 742m, 716m, 707s, 658m, 620s, 572m, 560m, 499m and 488m cm^−1^; ^1^H-NMR (400 MHz, CD_3_OD): δ = 6.10 (s, 1H, 3-H_b_), 3.84 (d, *J* = 1.1 Hz, 2H, 9-H), 2.30 (d, *J* = 18.7 Hz, 1H, 4-H_a_), 2.87 (t, *J* = 7.2 Hz, 1H, 3-H), 2.68 (d, *J* = 18.7 Hz, 1H, 4-H_b_), 1.94–1.84 (m, 1H, 7-H_a_), 1.66–1.57 (m, 1H, 7-H_b_) and 1.13 (t = 7.3 Hz, 3H, 8-H) ppm; ^13^C-NMR (100 MHz, CD_3_OD): δ = 175.3 (C-2), 174.9 (C-5), 105.7 (C-3b), 52.3 (C-3a), 49.4 (C-3), 37.6 (C-9), 33.6 (C-4), 21.6 (C-7) and 12.7 (C-8) ppm; MS (ESI, MeOH): *m/z* = 263.0 and 265.1 (47% and 48%, [M+H]^+^); analysis calcd. for C_9_H_11_BrO_4_ (263.09): C 41.09, H 4.21; found: C 40.79, H 4.32.

### 4.8. Hexyl (4-Ethyl-5-oxo-2,5-dihydrofuran-3-yl)acetate (15)

The reaction of **12** (1.0 g, 5.88 mmol) with thionyl chloride (2.0 mL, 10 mmol) and hexanol (20 mL, 160 mmol) was performed for 16 h under reflux followed by usual workup and chromatography (SiO_2_, *n*-hexane/ethyl acetate, 5:1) and produced **15** (1.45 g, 98%) as a colorless oil (pure enough for the next step); an analytical sample showed R_f_ = 0.28 (*n*-hexane/ethyl acetate, 5:1); IR (film): ν = 3630w, 2960s, 2935s, 2861m, 1756s, 1676m, 1654w, 1455m, 1380m, 1341m, 1267m, 1199s, 1178s, 1106m, 1090m, 1060m and 1036s cm^−1^; ^1^H-NMR (400 MHz, CDCl_3_): δ = 4.80 (s, 2H, 5-H), 4.12 (t, *J* = 6.7 Hz, 2H, 10-H), 3.46 (s, 2H, 8-H), 2.32 (q, *J* = 7.6 Hz, 6-H), 1.62 (q, 1H, *J* = 7.0 Hz, 11-H), 1.31 (br s, 6H, 12-H, 13-H, 14-H), 1.11 (t, *J* = 7.6 Hz, 3H, 7-H) and 0.90 (t, *J* = 6.7 Hz, 3H, 15-H) ppm; ^13^C-NMR (100 MHz, CDCl_3_): δ = 174.1 (C-9), 168.4 (C-2), 151.2 (C-4), 131.4 (C-3), 71.6 (C-5), 65.9 (C-10), 32.5 (C-8), 31.4 (C-11), 28.5 (C-12), 25.5 (C-13), 22.5 (C-14), 17.10 (C-6), 13.9 (C-15) and 12.6 (C-7) ppm; MS (ESI, MeOH): *m/z* = 255.4 (41%, [M+H]^+^); analysis calcd. for C_14_H_22_O_4_ (254.33): C 66.12, H 8.72; found: C 65.87, H 8.92.

### 4.9. (±)-Hexyl [(3RS, 4SR)-4-Ethyl-5-oxooxolan-3-yl] acetate [(±)-16]

The hydrogenation (1 atm) of **15** (1.23 g, 4.82 mmol) with Rh/Al_2_O_3_ (5%, 500 mg) in dry THF (15 mL) for 5 days followed by usual workup produced **(±)-16** (1.23 g, quant.) as a colorless oil; an analytical sample showed R_f_ = 0.52 (*n*-hexane/ethyl acetate, 3:1); IR (film): ν = 2960s, 2935s, 2861m, 1775s, 1735s, 1469m, 1375m, 1324m, 1215m, 1175s, 1111m, 1060m, 1030m and 987m cm^−1^; ^1^H-NMR (400 MHz, CDCl_3_): δ = 4.20 (ddd, *J* = 9.9, 5.9, 0.4 Hz, 1H, 5-H_a_), 4.11 (dd, *J* = 9.6, 3.7 Hz, 1H, 5-H_b_), 4.10 (t, *J* = 6.7 Hz, 2H, 10-H), 3.04–2.98 (m, 1H, 4-H), 2.56 (dd, *J* = 14.8, 8.0 Hz, 1H, 3-H), 2.47 (dd, *J* = 16.5, 4.7 Hz, 1H, 8-H_a_), 2.30 (dd, *J* = 16.5, 10.5 Hz, 1H, 8-H_b_), 1.85–1.75 (m, 1H, 6-H_a_), 1.61 (q, *J* = 7.0 Hz, 2H, 11-H), 1.53–1.42 (m, 1H, 6-H_b_), 1.42–1.30 (m, 6H, 12-H, 13-H, 14-H), 1.05 (t, *J* = 7.4 Hz, 3H, 7-H) and 0.91 (t, *J* = 6.7 Hz, 15-H) ppm; ^13^C-NMR (100 MHz, CDCl_3_): δ = 178.0 (C-9), 171.7 (C-2), 70.9 (C-5), 65.2 (C-10), 44.2 (C-3), 35. 2 (C-4), 32.0 (C-8), 31.4 (C-11), 28.5 (C-12), 25.6 (C-13), 22.5 (C-14), 18.5 (C-6), 14.0 (C-15) and 12.1 (C-7) ppm; MS (ESI, MeOH): *m/z* = 257.3 (41%, [M+H]^+^); analysis calcd. for C_14_H_24_O_4_ (256.34): C 65.60, H 9.44; found: C 65.41, H 9.67.

### 4.10. (+)-Hexyl 2-[(3S, 4R)-4-Ethyl-5-oxooxolan-3-yl]acetate [(+)-16] and (–)-2-[3R, 4S)-4-Ethyl-5-oxooxolan-3-yl] acetic acid [(–)-17]

In a pH-stat (Metrohm), a solution of **16** (1.26 g, 4.92 mmol) in distilled ater (80 mL) was stirred for 2 days at 22 °C with Lipase PS “Amano” (2.4 g), keeping the pH = 7 constant (27.35 mL, 0.1n NaOH). For workup, pH was adjusted to 6.5 with diluted with aqueous HCl (5%), and the reaction mixture was extracted with diethyl ether (3 × 200 mL). The organic phase was dried (MgSO_4_), the solvent was evaporated under diminished pressure, and the residue was subjected to chromatography (SiO_2_, *n*-hexane/ethyl acetate, 7:1 → 5:1 → 3:1 → 0:1) to yield **(+)-16** (605 mg, 48%) and **(–)-17** (398 mg, 42%).

Data for **(+)-16**: colorless oil; R_f_ = 0.52 (*n*-hexane/ethyl acetate, 3:1), [α]_D_ = +64.6° (*c* 1.0, CHCl_3_), [lit.: [42] [α]_D_ = +64.5° (*c* 1.26, CHCl_3_)]; ee > 99% by HPLC (Chiralcel OC; *n*-hexane/ethanol, 95:5, 0.6 mL/min, λ = 225 nm, t_R_ (+) = 22.5 min, t_R_ (–) = 124.7 min; Chiralpak AS (*n*-hexane/isopropanol, 98:2, 1 mL/min) t_R_(+) = 17.0 min and t_R_(–) = 22.3 min; IR (film): ν = 3566br m, 2971m, 1760s, 1700s, 1435m, 1406m, 1375m, 1326m, 1250m, 1177s, 1115s, 1056m, 1031m, 990s, 911m, 731m and 680m cm^−1^; ^1^H-NMR (400 MHz, CDCl_3_): δ = 8.93 (br s, 1H, CO_2_H), 4.34 (dd, *J* = 9.5, 5.1 Hz, 5-H_a_), 4.12 (dd, *J* = 9.5, 3.3 Hz, 1H, 5-H_b_), 3.06–2.95 (m, 1H, 4-H), 2.60 (dd, *J* = 14.8, 8.1 Hz, 1H, 3-H), 2.50 (dd, *J* = 105, 4.6 Hz, 1H, 8-H_a_), 2.35 (dd, *J* = 16.7, 10.5 Hz, 1H, 8-H_b_), 1.90–1.75 (m, 1H, 6-H_a_), 1.55–1.35 (m, 1H, 6-H_b_) and 1.07 (t, *J* = 7.4 Hz, 3H, 7-H) ppm; ^13^C-NMR (100 MHz, CDCl_3_): δ = 177.9 (C-9), 171.7 (C-2), 70.8 (C-5), 65.2 (C-10), 44.0 (C-3), 35.0 (C-4), 32.0 (C-8), 31.4 (C-11), 28.5 (C-12), 25.6 (C-13), 22.5 (C-14), 18.5 (C-6), 14.0 (C-15) and 12.1 (C-7) ppm; MS (ESI, MeOH): *m/z* = 257.3 (56%, [M+H]^+^); analysis calcd. for C_14_H_24_O_4_ (256.34): C 65.60, H 9.44; found: C 65.44, H 9.61.

Data for **(–)-17**: colorless oil [53,54]; R_f_ = 0.90 (methanol/ethyl acetate), 1:4); [α]_D_ = –87.1° (*c* 1.0, CHCl_3_); ee > 99% (by HPLC); IR (film): ν = 3566br m, 2970m, 1761s, 1700s, 1435m, 1404m, 1375m, 1250m, 1176s, 1118s, 1056m, 1030m, 990s, 911m, 730m and 680m cm^−1^; ^1^H-NMR (400 MHz, CDCl_3_): δ = 8.91 (br s, 1H, CO_2_H), 4.35 (dd, *J* = 9.5, 5.1 Hz, 1H, 5-H_a_), 4.12 (dd, *J* = 9.5, 3.3 Hz, 1H, 5-H_b_), 3.03–2.95 (m, 1H, 4-H), 2.58 (dd, *J* = 14.8, 8.1Hz, 1H, 3-H), 2.50 (dd, *J* = 10.5, 4.6 Hz, 1H, 8-H_a_), 2.35 (dd, *J* = 16.7, 10.5 Hz, 8-H_b_), 1.90–1.75 (m, 1H, 6-H_a_), 1.55–1.35 (m, 1H, 6-H_b_) and 1.05 (t, *J* = 7.4 Hz, 1H, 7-H) ppm; ^13^C-NMR (100 MHz, CDCl_3_): δ = 177.9 (CO_2_H), 177.0 (CO), 70.8 (C-5), 44.0 (C-3), 35.0 (C-4), 31.7 (C-8), 18.5 (C-6), 12.0 (Me); MS (ESI, MeOH): *m/z* = 173.2 (27%, [M+H]^+^); analysis calcd. for C_8_H_12_O_4_ (172.18): C 55.81, H 7.02; found: C 55.68, H 7.17.

### 4.11. (+)-2-[3S, 4R)-4-Ethyl-5-oxooxolan-3-yl] acetic acid [(+)-17]

In a pH-stat (Metrohm), a solution of **(+)-16** (6.7 g, 26.1 mmol) in dist. water (300 mL) was stirred for 8 h at 22 °C with PLE (300 μL, 30 mg/mL, Boehringer), keeping the pH = 7 constant (263.3 mL, 0.1n NaOH). For workup, pH was adjusted to 6.5 with diluted with aq. HCl (5%) and extracted with diethyl ether (3 × 200 mL). The organic phase was dried (MgSO_4_), the solvent was evaporated under diminished pressure, and the residue was subjected to chromatography (SiO_2_, *n*-hexane/ethyl acetate, 7:1 → 5:1 → 3:1 → 0:1) to yield **(+)-17** (4.32g, 96%); [α]_D_ = +87.9° (*c* 1.1, CHCl_3_) [lit.: [42[α]_D_ = +73.2–81.5° (CHCl_3_)]; R_f_ = 0.90 (methanol/ethyl acetate; ee > 99% (by HPLC); IR, ^1^H-NMR and ^13^C-NMR as well as MS (ESI, MeOH) identical to its enantiomer (vide supra); analysis calcd. for C_8_H_12_O_4_ (172.18): C 59.81, H 7.02; found: C 59.71, H 7.33.

### 4.12. (–) 2-[(3R, 4S)-4-Ethyl-5-oxooxolan-3-yl)-N-methoxy-N-methylacetamide [(–)-18]

To a solution of **(–)-17** (3.0 g, 17.4 mmol) in ethyl acetate (100 mL) at 0 °C, *N*-methylmorpholine (NMM, 1.9 mL, 17.45 mmol; in 25 mL dry ethyl acetate) was slowly added followed by the addition of isobutyl chloroformate (2.28 mL, 17.4 mmol, in 3 mL dry ethyl acetate). After stirring for 15 min, *N*,*O*-dimethylhydroxylamine hydrochloride (1.9 g, 19.2 mmol) was added followed by the addition of another portion NMM (1.9 mL, 17.45 mmol in 25 mL dry ethyl acetate). After stirring at 0 °C for 30 min and stirring at 23 °C for 1 day, the mixture was washed with water (5 mL), aq. citric acid (10%, 5 mL) and brine (10 mL), the organic layers were dried (MgSO_4_). The solvents were removed under diminished pressure, and the residue was purified by chromatography (SiO_2_, *n*-hexane/ethyl acetate 5:1 → 3:1 → 2:1 → 0:1) to yield **(–)-18** (3.18 g, 85%) was a colorless oil; R_f_ = 0.30 (*n*-hexane/ethyl acetate, 1:1); [α]_D_ = –113.8° (*c* 0.9, CHCl_3_), IR (film): ν = 2970m, 2942m, 2880m, 1770s, 1660s, 1650s, 1435m, 1420m, 1385m, 1260w, 1175s, 1117m, 1055m, 1005m, 985m and 946m cm^−1^; ^1^H-NMR (400 MHz, CDCl_3_): δ = 4.30 (dd, *J* = 9.3, 5.7 Hz, 1H, 5-H_a_), 4.10 (dd, *J* = 9.3, 2.7 Hz, 1H, 5-H_b_), 3.72 (s, 3H, NOMe), 3.21 (s, 3H, NMe), 3.15–3.00 (m, 1H, 4-H), 2.59 (dd, *J* = 7.3, 7.3 Hz, 1H, 3-H), 2.52 (dd, *J* = 10.6, 6.2 Hz, 1H, 8-H_a_), 2.40 (dd, *J* = 16.7, 10.6 Hz, 1H, 8-H_b_), 1.85–1.74 (m, 21H, 6-H_a_), 1.55-1.45 (m, 1H, 6-H_b_) and 1.05 (t, *J* = 7.4 Hz, 3H,7-H) ppm; ^13^C-NMR (100 MHz, CDCl_3_): δ = 178.2 (CON), 171.8 (CO), 71.3 (C-5), 61.3 (NOMe), 44.2 (C-3), 34.6 (C-4), 34.6 (NMe), 29.3 (C-8), 18.6 (C-6) and 12.1 (C-7) ppm; MS (ESI, MeOH): *m/z* = 216.4 (63%, [M+H]^+^); analysis calcd. for C_10_H_17_NO_4_ (215.25): C 55.80, H 7.96, N 6.51; found: C 55.61, H 8.13, N 6.38.

### 4.13. (+)2-[(3S, 4R)-4-Ethyl-5-oxooxolan-3-yl)-N-methoxy-N-methylacetamide [(+)-18]

Following the procedure given for its enantiomer, from **(+)-17** (6.0 g, 34.8 mmol) **(+)-18** (6.35 g, 84%) was obtained as a colorless oil; [α]_D_ = +114.1° (*c* 1.0, CHCl_3_); R_f_ = 0.30 (SiO_2_, *n*-hexane/ethyl acetate); IR (film), ^1^H-NMR, ^13^C-NMR and MS (ESI, MeOH) were identical to the enantiomer (vide supra); analysis calcd. for C_10_H_17_NO_4_ (215.25): C 55.80, H 7.96; found: C 55.63, H 8.17.

### 4.14. (–)-2-[(3R, 4S)-4-Ethyl-5-oxooxolan-3-yl]-acetaldehyde [(–)-19]

The reduction of **(–)-18** (1.96 g, 9.1 mmol) with lithium aluminium hydride (0.42 g, 11.1 mmol) in dry diethyl ether (200 mL) at -45 °C was followed by an additional stirring at 23 °C for 30 min and followed by usual aqueous workup, extraction (4 × 100 mL) and chromatography (SiO_2_, *n*-hexane/ethyl acetate, 5:1 → 2:1 → 1:1) to produce **(–)-19** (1.35 g, 95%) as a colorless oil; [56] R_f_ = 0.45 (SiO_2_, *n*-hexane/ethyl acetate, 1:1), [α]_D_ = –110.3° (*c* 0.7, CHCl_3_); IR (film): ν = 3419s, 2960s, 2879s, 1770m, 1731s, 1463m, 1382m, 1181m, 1155m, 1120s, 1070s, 1036s, 940s, 901m and 861m cm^−1^; ^1^H-NMR (400 MHz, CDCl_3_): δ = 9.82 (s, 1H, 9-H), 4.32 (ddd, *J* = 9.4, 5.8, 0.9 Hz, 1H, 5-H_a_), 4.00 (dd, *J* = 9.4, 3.2 Hz, 1H, 5-H_b_), 3.12–3.05 (m, 1H, 4-H), 2.66 (dd, *J* = 18.7, 4.5 Hz, 1H, 3-H), 2.56 (dd, *J* = 10.3, 3.7 Hz, 1H, 8-H_a_), 2.51 (dd, *J* = 10.3, 1.6 Hz, 1H, 8-H_b_), 1.85–1.77 (m, 1H, 6-H_a_), 1.50–1.34 (m, 1H, 6-H_b_) and 1.05 (t, *J* = 7.4 Hz, 3H, 7-H) ppm; ^13^C-NMR (100 MHz, CDCl_3_): δ = 199.2 (CHO), 177.7 (CO), 70.5 (C-5), 44.2 (C-3), 34.6 (C-4), 26.5 (C-8), 18.6 (C-6) and 12.0 (C-7) ppm; MS (ESI, MeOH): *m/z* = 157.1 (85%, [M+H]^+^); analysis calcd. for C_8_H_12_O_3_ (156.18): C 61.52, H 7.74; found: 61.39, H 8.04.

### 4.15. (+)-2-[(3S, 4R)-4-Ethyl-5-oxooxolan-3-yl]-acetaldehyde [(+)-19]

Following the procedure given for the enantiomer, **(+)-19** (0.67, 95%) was obtained as a colorless oil; R_f_ = 0.45 (SiO_2_, *n*-hexane/ethyl acetate, 1:1); [α]_D_ = +109.3° (*c* 0.3, CHCl_3_); IR (film), ^1^H-NMR, ^13^C-NMR and MS (ESI, MeOH) were identical to the enantiomer (vide supra); analysis calcd. for C_8_H_12_O_3_ (156.18): C 61.52, H 7.74; found: 61.35, H 8.11.

### 4.16. (–)-Pilocarpine [(–)-1]

A solution of methylamine in benzene (3.9 mL, 9.0 mmol) was added to a mixture of **(–)-19** (1.2 g, 7.7 mmol) and dry, finely powdered K_2_CO_3_ (3.2 g, 38.5 mmol) in dry DCM/benzene (150 mL, 1:1), and the reaction mixture was stirred for 3 h at 23 °C. The solvents were removed under diminished pressure, dry DCM (20 mL) was added, distilled off, and *p*-tosylmethylisocyanide (3.31 g, 38.5 mmol) and dry triethylamine (5.4 mL, 38.5 mmol) were added. After an additional stirring for 1 week, the solvents were removed and the residue purified by chromatography (SiO_2_, MeOH/DCM, 1.25% → 5%) to yield **(–)-1** (0.96 g, 60%) as a colorless oil; R_f_ = 0.60 (SiO_2_, DCM/MeOH/aq NH_4_OH (25%), 95:4:1); [α]_D_ = –114.7° (*c* 0.8, CHCl_3_), ee > 99% (by HPLC, Chiralcel OC, *n*-hexane/ethanol, 3:7, 0.3 mL/min, UV-detection = 215 nm; t_R_ = **(+)-1** 47.1 min, t_R_ = **(–)-1** = 52.32 min); IR (film): ν = 2965, 2880m, 1770s, 1655w, 1560w, 1505m, 1459m, 1425w, 1375m, 1316w, 1291w, 1270w, 1224m, 1176s, 1109m, 1050m, 1024m and 980m cm^−1^; ^1^H-NMR (400 MHz, CDCl_3_): δ = 7.42 (s, 1H, N=CH-N-CH_3_), 6.81 (s, 1H, C=CH-N), 4.20 (dd, *J* = 9.2, 5.6 Hz, 1H, CH_2_-O), 4.10 (dd, *J* = 9.2, 2.7 Hz, 1H, -CH_2_-O), 3.59 (s, 3H, N-CH_3_), 2.85–2.75 (m, 1H, -CH-), 2.71 (dd, *J* = 15.3, 3.9 Hz, 1H, -CH_2_-), 2.64 (dd, *J* = 8.4, 6.8 Hz, 1H, CO-CH), 2.41 (dd, *J* = 15.3, 12.0 Hz, 1H, -CH_2_-), 1.95–1.80 (m, 1H, ethyl), 1.68–1.50 (m, 1H, ethyl) and 1.12 (t, *J* = 7.5 Hz, 3H, CH_3_) ppm; ^13^C-NMR (100 MHz, CDCl_3_): δ = 177.9 (CO), 138.3 (N=CH-N), 128.6 (=CH), 127.1 (=CH-N), 69.9 (CH_2_-O), 44.9 (CO-CH), 37.39 (-CH-), 31.2 (N-CH_3_), 21.4 (-CH_2_-), 18.3 (CH_2_) and 12.2 (CH_3_) ppm; MS (ESI, MeOH): *m/z* = 209.2 (76%, [M+H]^+^); analysis calcd. for C_11_H_16_N_2_O_2_ (208.26): C 63.44, H 7.74, N 13.45; found: C 63.21, H 7.96, N 13.29.

### 4.17. (+)-Pilocarpine [(+)-1]

Following the procedure given for the synthesis of its enantiomer, **(+)-1** (1.92 g, 59%) was obtained as a colorless oil; R_f_ = 0.60 (SiO_2_, DCM/MeOH/aq NH_4_OH (25%), 95:4:1); [α]_D_ = +115.7° (*c* 0.6, CHCl_3_), ee > 99% (by HPLC, Chiralcel OC, *n*-hexane/ethanol, 3:7, 0.3 mL/min, UV-detection λ = 215 nm; t_R_ = **(+)-1** 47.1 min, t_R_ = **(–)-1** = 52.32 min); IR (film), ^1^H-NMR, ^13^C-NMR and MS (ESI, MeOH) were identical to the enantiomer (vide supra); analysis calcd. for C_11_H_16_N_2_O_2_ (208.26): C 63.44, H 7.74, N 13.45; found: C 63.31, H 7.98, N 13.32

## Figures and Tables

**Table 1:** Crystallographic data for compound **13**.

Compound	13
Molecular formula	C_9_H_11_BrO_4_
Formula weight /g·mol^–1^	263.09
Crystal system	monoclinic
Space group	P 2_1_/n
*a* /pm	633.6(3)
*b* /pm	1269.4(3)
c /pm	1208.9(3)
*β*/°	94.30(4)°
Cell volume /nm^3^	0.9696(6)
Molecules per cell *Z*	4
Calc. density *ρ* /g·cm^–3^	1.802
*μ* (Mo-*K*a) /mm^–1^	4.225
Crystal size/mm	0.6 × 0.56 × 0.4
Diffractometer	STOE STADI IV
*T* /K	293(2)
θ range /°	2.33–25.96
Absorption correction	empirical
Reflections collected	5393
Reflections unique	1887
Reflections with *F_o_* > 4σ(*F_o_*)	1680
Completeness of dataset /%	99.9
*R*_int_	0.0435
Parameters	88
*R*_1_ (*I* > 2σ(*I*))	0.0379
*wR*_2_ (all data)	0.0998
GooF (*F*^2^)	1.080

## Data Availability

The data presented in this study are available on request from the corresponding author.

## Sample Availability

Samples of the compounds are available from the authors.
